# Effects of Transcutaneous Electrical Acupoint Stimulation on Stress Response during Intubation and Extubation in Patients Undergoing Video-Assisted Thoracoscopic Surgery: A Prospective, Randomized Controlled Trial

**DOI:** 10.1155/2021/1098915

**Published:** 2021-09-30

**Authors:** Zhiyan Yu, Yuying Zhang, Huan Zhang, Xue Zhao, Hua Wei, Shuangliang He, Jianming Liu, Tiejun Liu

**Affiliations:** ^1^Department of Anesthesiology, North China University of Science and Technology Affiliated Hospital, Tangshan 063000, China; ^2^Department of Geriatrics, North China University of Science and Technology Affiliated Hospital, Tangshan 063000, China; ^3^Department of Neurosurgery, Tangshan People's Hospital, North China University of Science and Technology, Tangshan 063000, China; ^4^Department of Laboratory, Tangshan Maternal and Child Health Hospital, North China University of Science and Technology, Tangshan 063000, China; ^5^Department of Anesthesiology, Tangshan People's Hospital, North China University of Science and Technology, Tangshan 063000, China; ^6^Department of Thoracic Surgery, Tangshan People's Hospital, North China University of Science and Technology, Tangshan 063000, China

## Abstract

**Objective:**

The study aimed to evaluate the effect of transcutaneous electrical acupoint stimulation (TEAS) on the stress response during intubation and extubation in patients undergoing video-assisted thoracoscopic surgery (VATS).

**Methods:**

122 patients undergoing VATS lobectomy were randomly divided into two groups: the TEAS group (*n* = 62) and the control group (*n* = 60). Patients in the TEAS group underwent electroacupuncture stimulation of bilateral Neiguan (PC6), Hegu (L14), Lieque (LU7), and Chize (LU5) acupoints from 30 min before anesthesia to the end of surgery. The patients in the control group did not undergo stimulation. The primary endpoints were the hemodynamic parameters and plasma concentrations of epinephrine, norepinephrine, and cortisol. The secondary endpoints were the consumption of remifentanil and propofol, Ramsay sedation score and arousal time, extubation quality score, and postoperative complications.

**Results:**

The hemodynamic variables and plasma concentrations of epinephrine, norepinephrine, and cortisol during intubation and extubation were lower in the TEAS group at T1, T3, and T4 compared with the control group. TEAS led to a reduction in the consumption of remifentanil (*P* < 0.01), as well as a reduction in the incidence of postoperative complications. The extubation quality score was lower (*P* < 0.01) while the Ramsay sedation score was higher (*P* < 0.01) in the TEAS group than in the control group. However, the arousal time and consumption of propofol were not significantly different between the two groups.

**Conclusion:**

TEAS can maintain hemodynamic stability, reduce the stress response during intubation and extubation, improve the quality of anesthesia recovery, and decrease the incidence of postoperative complications in patients undergoing VATS.

## 1. Introduction

When the body is stimulated by a certain stress, the excitability of sympathetic nerves increases, and the activity of the hypothalamus-pituitary-adrenal cortex axis increases. This specific defense response is called stress response. When a stress response occurs, a series of stress factors such as C-reactive protein, cortisol, norepinephrine, and epinephrine are released into the blood in large quantities, which can increase heart rate, blood pressure, and neuromuscular excitability [1, 2].

After induction of general anesthesia, tracheal intubation and other related operations can cause the body to produce a strong stress response, hemodynamics can fluctuate sharply, and even adverse events such as malignant arrhythmia can occur [3, 4]. For high-risk patients, the increase in adrenaline, norepinephrine, and dopamine in the body may lead to an increase in perioperative mortality. Tracheal extubation is usually performed during the emergence phase after stopping anesthetics. Significant systemic stress responses to pain and airway stimulation can occur during this period, including hypertension, tachycardia, and increased intracranial hypertension. This induces respiratory reactions, such as breath-holding, restlessness, choking, and bronchospasm [5, 6]. Therefore, inhibiting the strong stress response caused by tracheal intubation and extubation is particularly important to ensure the stability of hemodynamics, avoid the occurrence of related cardiovascular events, and ensure the safety and stability of patients through the operation period.

Transcutaneous electrical acupoint stimulation (TEAS) is a noninvasive form of electrical acupoint stimulation. Different from traditional acupuncture intervention, i.e., inserting a needle into acupoint and applying manual stimulation including acupuncture and electroacupuncture, the stimulation onto acupoint is transmitted through electricity transmitted through the surface electrodes [7, 8]. Previous studies have proven that TEAS can be successfully applied for various aspects of the perioperative period, such as relieving pain and nausea and vomiting after surgery, reducing intraoperative anesthetic consumption, and improving postoperative recovery [9, 10]. Bai et al. [11] reported that TEAS restrains the stress response during extubation and improves the quality of recovery after general anesthesia in elderly patients undergoing elective supratentorial craniotomy. However, the effects of TEAS on stress response during intubation and extubation, particularly in patients undergoing video-assisted thoracoscopic surgical (VATS), has not been explored. Therefore, this study aimed to explore the combined effect of TEAS on stress response during intubation and extubation in patients undergoing VATS.

## 2. Methods

### 2.1. Participants

The study participants had an American Society of Anesthesiologists physical state of I or II and were scheduled for VATS lobectomy under general anesthesia. The exclusion criteria were as follows: (1) participants with obvious functional abnormalities of the heart, lung, liver, or kidney; (2) participants in whom the target acupoint stimulation site was infected, who had trauma, or for whom TEAS implementation was not recommended for other reasons; (3) participants who were unable to communicate with doctors effectively and cooperate with them to complete the trial. This research was approved by the Ethics Committee of the Tangshan People's Hospital, North China University of Science and Technology. All participants provided written consent prior to randomization.

### 2.2. Randomization and Blinding

Patients were randomly assigned to either the TEAS group or the control group in a 1 : 1 ratio using a computer-generated random number sequence. The group assignment was sealed in consecutively numbered opaque envelopes. The study was designed as a single-blind study in which random codes and corresponding treatment measures were only known from acupuncture. Patients, caregivers, researchers, and senior researchers were unaware of the randomization pattern. Patients who did not receive electrical point stimulation were not informed about the assignment of possible reactions to control electroacupuncture procedures.

### 2.3. Standardized Anesthesia

Propofol medium and long-chain fat emulsion is pumped continuously at the rate of 4∼8 mg/kg/h from the beginning of the preparation. Remifentanil is diluted with normal saline to 20 ug/ml and pumped at the same time at a rate of 8–10 ug/kg/h. Adjust the speed of propofol and remifentanil according to hemodynamic parameters and BIS to stabilize BIS at 40∼55. Muscle relaxants are added regularly during the operation. Reduce the dosage of propofol after resuming ventilation of both lungs. No opioids other than the infused remifentanil were given during the operation. TEAS and all anesthetic infusions were stopped 5 minutes before the end of surgery.

### 2.4. Intervention

TEAS was conducted by an experienced acupuncturist. The bilateral Hegu (LI4), Neiguan (PC6), Lieque (LU7), and Chize (LU5) acupoints were selected as the acupuncture points based on the traditional Chinese medical theory. Electrical simulation was performed using the Hwato SDZ-II Acupoint Stimulator (Suzhou Medical Appliances Co. Ltd., Suzhou, China).

The participants were divided into two groups: TEAS group and control group. The TEAS group received acupoint electrical stimulation at 30 min before the induction of anesthesia. The stimulus frequency is 2/100 Hz dense-disperse wave. The optimal intensity ranged from 6 to 15 mA, and it was regulated to maintain a slight twitching of the regional muscles based on individual maximum tolerance. None of the participants showed signs of intense pain or communicated discomfort. The stimulus was maintained until the end of surgery. The control group had electrode pads applied but received no electrical stimulation.

### 2.5. Measurements

The primary endpoints were the stress response during extubation, assessed using hemodynamic indices (MAP, HR), and the plasma concentrations of epinephrine, norepinephrine, and cortisol at 30 min before the induction of anesthesia (T0), immediately after intubation (T1), 5 min after intubation (T2), immediately after extubation (T3), and 5 min after extubation (T4). The secondary endpoints were the consumption of remifentanil and propofol, Ramsay sedation score and arousal time, extubation quality score, and postoperative complications. Postoperative complications included hypertension, tachycardia, cough, agitation, and postoperative nausea and vomiting (PONV).

The Ramsay sedation score is a 6-point scale, of which 1 point represents anxiety; 2 points, calmness, cooperation, and orientation; 3 points, individual responses to instructions; 4 points, fall asleep and react quickly when tapped between the brows or called aloud; 5 points, fall asleep and be unresponsive when tapped lightly between eyebrows or called aloud; and 6 points, unresponsive to stimuli, that is, deep sleep or anesthesia.

The extubation quality score is 5 points, of which 1 point means no cough; 2 points, unobstructed extubation and minimal cough (1–2 times); 3 points, moderate cough (3–4 times); 4 points, severe cough (5–10 times) and strain; 5 points, poor extubation and high discomfort (laryngospasm and cough >10 times).

### 2.6. Statistical Analysis

Continuous variables were expressed as mean ± standard deviation (SD) and processed with Student's *t* test. Categorical variables were expressed as numbers (percentage) and processed with Chi-square test. The MAP, HR, and plasma concentrations of epinephrine, norepinephrine, and cortisol over multiple time points were compared using repeated-measures analysis of variance followed by a Bonferroni correction for multiple comparisons. A value of *P* < 0.05 was considered statistically significant. Statistical analysis was performed by using SPSS version 19.0 (SPSS Inc., Chicago, IL, USA).

## 3. Results

### 3.1. Trial Completion Condition

**Figure 1:**
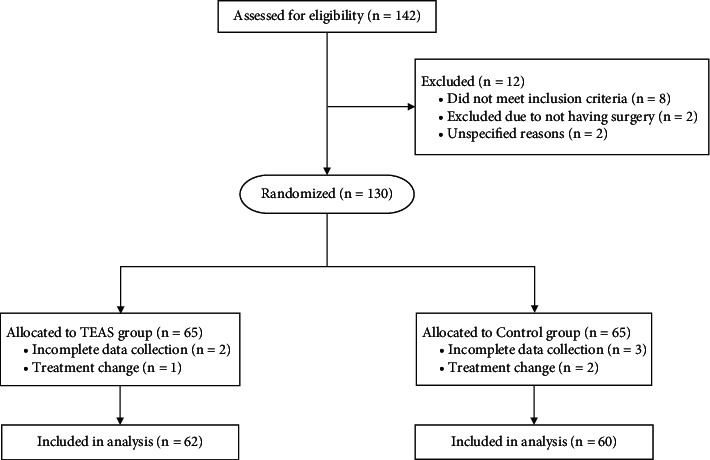
Flow chart of the study. TEAS: transcutaneous electrical acupoint stimulation.

This trial recruited 142 patients, among whom 131 patients were eligible. However, only 122 participants (62 in the TEAS group and 60 in the control group) were enrolled in this study (Figure 1). The baseline characteristics and demographic data were similar between both groups (Table 1).

### 3.2. Hemodynamic Variables at Different Time Points

**Figure 2:**
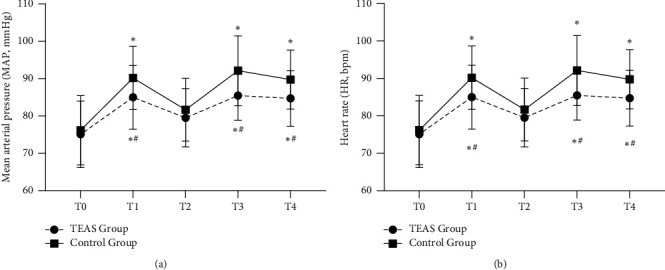
Heart rate and mean arterial pressure changes in the two groups. (a) Mean arterial pressure. (b) Heart rate. The patients in the TEAS group received electroacupuncture stimulation of bilateral Hegu (LI4), Neiguan (PC6), Lieque (LU7), and Chize (LU5) acupoints from 30 min before anesthesia to the end of surgery. The patients in the control group were not given the stimulation. TEAS, transcutaneous electrical acupoint stimulation; T0, 30 min before the induction of anesthesia; T1, immediately after intubation; T2, 5 min after intubation; T3, immediately after extubation; T4, 5 min after extubation. Data are presented as mean ± standard deviation ($\bar{x}\pm \text{s}$). ^*∗*^*P* < 0.05 versus T0; ^#^*P* < 0.05 versus the control group.

Compared with the value at T0, the MAP and HR at T1, T3, and T4 in both groups increased significantly (*P* < 0.05). MAP and HR in TEAS group at T1, T3, and T4 were lower than those in control group (*P* < 0.05) (Figures 2(a) and 2(b)).

### 3.3. Plasma Concentrations of Epinephrine, Norepinephrine, and Cortisol

**Figure 3:**
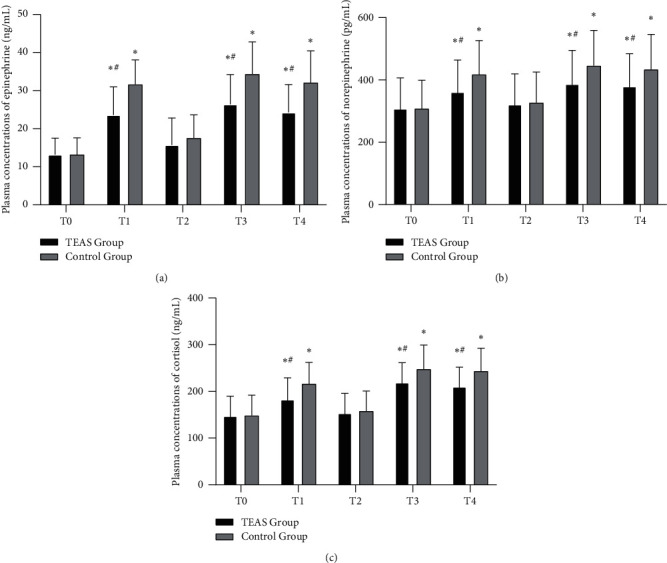
Plasma concentrations of epinephrine, norepinephrine, and cortisol in the two groups. (a) Plasma concentrations of epinephrine; (b) plasma concentrations of norepinephrine; (c) plasma concentrations of cortisol. The patients in the TEAS group received electroacupuncture stimulation of bilateral Hegu (LI4), Neiguan (PC6), Lieque (LU7), and Chize (LU5) acupoints from 30 min before anesthesia to the end of surgery. The patients in the control group were not given the stimulation. TEAS, transcutaneous electrical acupoint stimulation; T0, 30 min before the induction of anesthesia; T1, immediately after intubation; T2, 5 min after intubation; T3, immediately after extubation; T4, 5 min after extubation. Data are presented as mean ± standard deviation ($\bar{x}\pm \text{s}$). ^*∗*^*P* < 0.05 versus T0; ^#^*P* < 0.05 versus the control group.

Compared with T0, the plasma concentrations of epinephrine, norepinephrine, and cortisol in the two groups were significantly higher at T1, T3, and T4 (*P* < 0.05). All plasma concentrations in the TEAS group were lower than those in the control group (*P* < 0.05) (Figures 3(a)–3(c)).

### 3.4. Comparison of Perioperative Data between the Two Groups

Remifentanil consumption (*P* < 0.01) and extubation quality score (*P* < 0.01) in TEAS group were lower than those in control group. The Ramsay sedation score in TEAS group was higher than that in control group (*P* < 0.01). However, there was no significant difference in propofol consumption and arousal time between the two groups.

The incidence of tachycardia (*P*=0.02), cough (*P*=0.04), and PONV (*P*=0.05) was significantly lower in the TEAS group than in the control group. Although there was no significant difference in the incidence of hypertension and agitation between the two groups, fewer patients in the TEAS group had hypertension and/or agitation (Table 2).

## 4. Discussion

This prospective, single-blind, RCT showed that TEAS is a safe and feasible technique on stress response during intubation and extubation in patients undergoing VATS. The primary outcome showed that the hemodynamic variables and plasma concentrations of epinephrine, norepinephrine, and cortisol during intubation and extubation were lower in the TEAS group at T1, T3, and T4 compared with the control group. Other outcomes indicated TEAS was associated with significantly decreased consumption of remifentanil, lower extubation quality score, higher Ramsay sedation score, and lower incidence of postoperative complications. However, the arousal time and consumption of propofol were not significantly different between the two groups.

Many studies have explored ways to effectively alleviate the stress responses, agitation, and discomfort caused by intubation and extubation. Vasoactive drugs, such as esmolol, help to inhibit the cardiovascular response caused by tracheal extubation. However, they can only improve tachycardia and hyperactivity, cannot inhibit the strong stimulation of the tracheal tube to the trachea, and cannot effectively prevent agitation and discomfort during extubation [12]. Long-acting opioid analgesics, such as fentanyl, sufentanil, and dezocine, have been used to suppress stress response and discomfort during periextubation and have shown certain effects. However, due to the longer elimination half-life and the longer context-sensitive half-life, the controllability of long-acting opioid analgesics is poor. Fentanyl can not only alleviate stress responses, but also can cause respiratory depression and delay recovery [13].

The theory of traditional Chinese medicine believes that the main reason for the respiratory response of patients with tracheal intubation and extubation during perioperative period is the stimulation of the pulmonary system; therefore, this study chose acupuncture points that maintain the balance of energy flow in the pulmonary system and provide analgesia to inhibit the stress response during intubation and extubation. Specifically, the Hegu (LI4) acupoint belongs to the Large Intestine Meridian of Hand-Yangming and has been reported to be closely related to analgesia and sedation [14]. A clinical study has shown that in patients undergoing supratentorial craniotomy, stimulation of Hegu (LI4) can reduce the dosage of sevoflurane and shorten the time required to resume spontaneous breathing, extubation, and eye opening [15]. Neiguan (PC6) point belongs to the Hand-Jueyin Pericardium Meridian, which is reported to have a sedative effect and can reduce postoperative PONV [16]. Xu et al. [17] suggested that electroacupuncture stimulation of L14 and PC6 can adjust the hemodynamic stability of elderly patients during laparoscopic cholecystectomy and can effectively reduce the fluctuation of blood pressure and HR. Both Lieque (LU5) and Chize (LU7) belong to the Lung Meridian of Hand-Taiyin. It is reported that they have analgesic effect and are beneficial to the treatment of cough, headache, and intractable hiccups [18]. In this study, we selected Hegu (LI4), Neiguan (PC6), Lieque (LU7), and Chize (LU5) as acupuncture points to improve stress response.

Tracheal intubation and extubation mainly excite sympathetic nerves and stimulate the adrenal medulla to secrete a large amount of catecholamines into the blood to cause stress response, in order to improve the body's ability to deal with injuries [19]. As a result, the levels of epinephrine and norepinephrine can reflect the level of stress response. The release of catecholamines into the blood has a great influence on the hemodynamics of patients. Intense contraction of peripheral arteries and increased myocardial contractility increase blood pressure and HR [20]. Therefore, in this study, MAP and HR were used as hemodynamic variables to evaluate the stress response. When the body through the discharge of HPA axis and stress reaction secretes a large number of glucocorticoids, the concentration of cortisol increases rapidly. If the stress response continues, the cortisol concentration will remain high for a long time [21]. Plasma cortisol levels increased after induction of anesthesia and peaked on the first postoperative day. If there are no other complications, cortisol levels decrease to near preoperative levels about 48 to 72 h after surgery. Cortisol is an important stress medium in perioperative period [22]. In our study, the hemodynamic variables and plasma concentrations of epinephrine, norepinephrine, and cortisol increased to varying degrees in both groups compared to baseline values at T0. Tracheal intubation and extubation caused hemodynamic changes and stress response, but the degree of the two groups was different. The TEAS group had lower hemodynamic variables and plasma concentrations of epinephrine, norepinephrine, and cortisol at T1, T3, and T4 compared to the control group. A study has shown that TEAS can reduce the stress response during extubation in elderly patients undergoing elective supratentorial craniotomy, which is consistent with our results. Our results show that TEAS can reduce the stress response of patients during intubation and extubation by maintaining hemodynamic stability and reducing the plasma concentrations of epinephrine, norepinephrine, and cortisol. However, at T2, there was no significant difference in hemodynamic variables and plasma concentrations of epinephrine, norepinephrine, and cortisol between the two groups, which may be related to the adjustment of anesthesia depth after intubation.

Although there was no significant difference in propofol consumption and arousal time between the two groups in our study, we found significant reductions in remifentanil consumption and extubation quality scores in the TEAS group. The Ramsay sedation score was significantly higher in the TEAS group. This finding suggests that TEAS has an analgesic and sedative actions inducing the release of endogenous opioids. However, while maintaining the same anesthetic depth, the concentration of propofol and the perioperative MAP value cannot be reduced; thus, anesthetic depth has no effect on the arousal time. The incidences of tachycardia, cough, and PONV were significantly lower in the TEAS group than in the control group. Although the incidence of hypertension and agitation in the two groups was not significantly different, fewer patients developed hypertension and/or agitation in the TEAS group.

Several mechanisms have been proposed for the therapeutic efficacy of acupuncture, electroacupuncture, or TEAS. First, TEAS directly stimulates type I and type II afferent nerve fibers in the muscle, and the impulse was transmitted to the anterior lateral cord of the notochord through these fibers and then stimulated the spinal cord to release *β*-endorphins, enkephalins, and strong kephalins, blocking the transmission of pain impulse in the presynaptic space, thus blocking the upward conduction of pain signals in the spinal-thalamic tract [23]. Second, acupuncture can also regulate the levels of ACTH and cortisol in plasma after operation and reduce the level of corticotropin-releasing hormone messenger ribonucleic acid (CRHmRNA) in hypothalamus after operation, thereby inhibiting the overexcitability of the HPA axis in the stress response to a certain extent and reducing the secondary damage caused by the stress response to the body [24]. Finally, perioperative acupuncture can inhibit the increase of interleukin-6 (IL-6) and IL-10 in serum after surgery, control the fluctuation range of plasma malondialdehyde, and inhibit the decrease of plasma NO level after surgery, so as to reduce postoperative inflammatory reaction and oxidative stress reaction [25].

There are some limitations in this study. First, we did not evaluate the presence of immune humoral factors during intubation and extubation. In fact, the concentration of immune humoral factors should be considered an outcome of the present study. The clinical relationship between immune humoral factors and the perioperative stress response has not been fully elucidated. However, a large number of clinical studies have shown that abnormal coagulation, fibrinolysis, and hyperthermic reactions in patients after a perioperative stress response are closely related to the release of immune humoral factors. Second, the secretion of cortisol follows the circadian rhythm, i.e., the cortisol levels are the highest in the morning (06 : 00–08 : 00) and the lowest during the night (00 : 00–02 : 00). In this study, all patients had different admission times; i.e., there may have been some differences in the basic plasma cortisol values, which may have affected the experimental results to a certain extent. Third, the study period for the primary endpoint was short (5 min after intubation and 5 min after extubation). In fact, the stress response caused by intubation and extubation may affect patients' disease progression and prognosis for a long time after surgery and may even increase postoperative mortality.

## 5. Conclusion

In conclusion, our study shows that TEAS maintains hemodynamic stability, reduces the stress response during intubation and extubation, improves the quality of anesthesia recovery, and decreases the incidence of postoperative complications in patients undergoing VATS.

## Figures and Tables

**Table 1:** Baseline characteristics of the patients.

Variables	TEAS group (*n* = 62)	Control group (*n* = 60)	*P* value

Gender (*n* (%))			
Male	33 (53.2)	29 (48.3)	0.437
Female	29 (46.8)	31 (51.7)	
Age (years)	64.32 ± 10.21	62.14 ± 11.34	0.595
Weight (kg)	59.45 ± 11.43	58.42 ± 11.92	0.514
BMI (kg/m^2^)	23.63 ± 4.01	22.37 ± 5.74	0.419
ASA rating (*n* (%))			
I	43 (69.4)	45 (75.0)	0.419
II	19 (30.6)	15 (25.0)	
Operation time (min)	165.26 ± 17.47	168.48 ± 15.38	0.641
Anesthesia time (min)	187.47 ± 21.62	188.58 ± 25.27	0.818
Side of surgery (*n* (%))			
Left side	31 (50.0)	31 (51.7)	
Right side	31 (50.0)	29 (48.3)	0.841
TNM stage (*n* (%))			
I	35 (56.5)	33 (55.0)	0.742
II	27 (43.5)	27 (45.0)	

*Note.* Patients in the TEAS group received electroacupuncture stimulation of bilateral Hegu (LI4), Neiguan (PC6), Lieque (LU7), and Chize (LU5) acupoints from 30 min before anesthesia to the end of surgery. The patients in the control group did stimulation. TEAS: transcutaneous electrical acupoint stimulation; ASA: American Society of Anesthesiologists; BMI: body mass index; TNM: tumor, node, and metastasis.

**Table 2:** Comparison of perioperative data between the two groups.

	TEAS group (*n* = 62)	Control group (*n* = 60)	*P* value

Intraoperative period			
Remifentanil consumption (*μ*g/kg/min)	0.06 ± 0.03	0.09 ± 0.04	<0.01
Propofol consumption (mg/kg/h)	6.8 ± 2.3	7.3 ± 2.2	0.44
Ramsay sedation score (score)	3.85 ± 1.29	1.57 ± 1.04	<0.01
Arousal time (min)	11.54 ± 2.07	11.38 ± 2.13	0.15
Extubation quality score (score)	1.45 ± 1.03	3.75 ± 1.12	<0.01

Postoperative period			
Hypertension (*n* (%))	14 (22.6)	21 (35.0)	0.23
Tachycardia (*n* (%))	15 (24.2)	32 (53.3)	0.02
Cough (*n* (%))	17 (27.4)	30 (50.0)	0.04
Agitation (*n* (%))	9 (14.5)	17 (28.3)	0.17
PONV (*n* (%))	21 (33.9)	37 (61.7)	0.05

*Note.* Patients in the TEAS group received electroacupuncture stimulation of bilateral Hegu (LI4), Neiguan (PC6), Lieque (LU7), and Chize (LU5) acupoints from 30 min before anesthesia to the end of surgery. Patients in the control group did not receive stimulation. TEAS: transcutaneous electrical acupoint stimulation; PONV: postoperative nausea and vomiting.

## Data Availability

The datasets used and/or analysed during the current study are available from the corresponding author on reasonable request.

## References

[1] Khoo B., Boshier P. R., Freethy A. (2017). Redefining the stress cortisol response to surgery. *Clinical Endocrinology*.

[2] Smith S. M., Vale W. W. (2006). The role of the hypothalamic-pituitary-adrenal axis in neuroendocrine responses to stress. *Dialogues in Clinical Neuroscience*.

[3] Barak M., Ziser A., Greenberg A., Lischinsky S., Rosenberg B. (2003). Hemodynamic and catecholamine response to tracheal intubation: direct laryngoscopy compared with fiberoptic intubation. *Journal of Clinical Anesthesia*.

[4] El-Shmaa N. S., El-Baradey G. F. (2016). The efficacy of labetalol vs dexmedetomidine for attenuation of hemodynamic stress response to laryngoscopy and endotracheal intubation. *Journal of Clinical Anesthesia*.

[5] Artime C. A., Hagberg C. A. (2014). Tracheal extubation. *Respiratory Care*.

[6] Yao Z. Y., Jia Z., Xie Y. H. (2017). Analgesic effect of dezocine in different doses on elderly patients undergoing abdominal operation under general anesthesia and its influence on stress response to postoperative tracheal extubation. *European Review for Medical and Pharmacological Sciences*.

[7] Gao F., Zhang Q., Li Y. (2018). Transcutaneous electrical acupoint stimulation for prevention of postoperative delirium in geriatric patients with silent lacunar infarction: a preliminary study. *Clinical Interventions in Aging*.

[8] Yuan J., Wu Y., Li J. Y., Zhang Li, Chen Xi, Chen He-xiang (2015). Protection of transcutaneous acupoint electrical stimulation for brain injury undergoing intervention: a clinical observation. *Zhongguo Zhong Xi Yi Jie He Za Zhi*.

[9] Huang S., Peng W., Tian X. (2017). Effects of transcutaneous electrical acupoint stimulation at different frequencies on perioperative anesthetic dosage, recovery, complications, and prognosis in video-assisted thoracic surgical lobectomy: a randomized, double-blinded, placebo-controlled trial. *Journal of Anesthesia*.

[10] Liu X., Li S., Wang B., An L., Ren X., Wu H. (2015). Intraoperative and postoperative anaesthetic and analgesic effect of multipoint transcutaneous electrical acupuncture stimulation combined with sufentanil anesthesia in patients undergoing supratentorial craniotomy. *Acupuncture in Medicine*.

[11] Bai W.-Y., Yang Y.-C., Teng X.-F., Wan Y.-X., Wei W., Zhu J.-C. (2018). Effects of transcutaneous electrical acupoint stimulation on the stress response during extubation after general anesthesia in elderly patients undergoing elective supratentorial craniotomy: a prospective randomized controlled trial. *Journal of Neurosurgical Anesthesiology*.

[12] Kovac A. L., Masiongale A. (2007). Comparison of nicardipine versus esmolol in attenuating the hemodynamic responses to anesthesia emergence and extubation. *Journal of Cardiothoracic and Vascular Anesthesia*.

[13] Safavi M., Honarmand A. (2008). Attenuation of cardiovascular responses to laryngoscopy and tracheal intubation--intravenous sufentanil vs pethidine. *Middle East Journal of Anesthesiology*.

[14] Shen Y. F., Younger J., Goddard G., Mackey S (2009). Randomized clinical trial of acupuncture for myofascial pain of the jaw muscles. *Journal of Orofacial Pain*.

[15] Yang C., An L., Han R., Lin S., Kang X., Wang B. (2012). Effects of combining electro-acupuncture with general anesthesia induced by sevoflurane in patients undergoing supratentorial craniotomy and improvements in their clinical recovery profile & blood enkephalin. *Acupuncture & Electro-Therapeutics Research*.

[16] Lv J.-q., Feng R.-z., Li N. (2013). P6 acupoint stimulation for prevention of postoperative nausea and vomiting in patients undergoing craniotomy: study protocol for a randomized controlled trial. *Trials*.

[17] Xu S. Y., Huang Y. S., Chen X. W. (2016). Effect of transcutaneous acupoint electrical stimulation on stress reactions in laparoscopic cholecytectomy (LC) under anesthesia. *Zhonghua Zhong Yi Yao Xue Kan*.

[18] Fang X. J., Dong L. (2015). Acupuncture at Chize (LU5) for 23 cases of intractable hiccup. *Zhongguo Zhen Jiu*.

[19] Yu H.-C., Geng W.-J., Tang H.-L (2009). Effect of transcutaneous electrical acupoint stimulation on intratracheal extubation stress response in general anesthesia of patients with Breast Cancer Undergoing Modified Radical Mastectomy. *Zhongguo Zhong Xi Yi Jie He Za Zhi*.

[20] Manne G., Upadhyay M., Swadia V. (2014). Effects of low dose dexmedetomidine infusion on haemodynamic stress response, sedation and post-operative analgesia requirement in patients undergoing laparoscopic cholecystectomy. *Indian Journal of Anaesthesia*.

[21] Kutlesic M. S., Kutlesic R. M., Mostic-Ilic T. (2016). Attenuation of cardiovascular stress response to endotracheal intubation by the use of remifentanil in patients undergoing Cesarean delivery. *Journal of Anesthesia*.

[22] Prete A., Yan Q., Al-Tarrah K. (2018). The cortisol stress response induced by surgery: a systematic review and meta-analysis. *Clinical Endocrinology*.

[23] Zhang R., Lao L., Ren K., Berman B. M. (2014). Mechanisms of acupuncture-electro-acupuncture on persistent pain. *Anesthesiology*.

[24] Oh J.-Y., Kim Y.-K., Kim S.-N. (2018). Acupuncture modulates stress response by the mTOR signaling pathway in a rat post-traumatic stress disorder model. *Scientific Reports*.

[25] Zijlstra F. J., van den Berg-de Lange I., Huygen F. J. P. M., Klein J. (2003). Anti-inflammatory actions of acupuncture. *Mediators of Inflammation*.

